# Distinct Cleavage Properties of Cathepsin B Compared to Cysteine Cathepsins Enable the Design and Validation of a Specific Substrate for Cathepsin B over a Broad pH Range

**DOI:** 10.1021/acs.biochem.3c00139

**Published:** 2023-07-17

**Authors:** Michael C. Yoon, Von Phan, Sonia Podvin, Charles Mosier, Anthony J. O’Donoghue, Vivian Hook

**Affiliations:** † Skaggs School of Pharmacy and Pharmaceutical Sciences, University of California, La Jolla, San Diego, California 92093, United States; ‡ Biomedical Sciences Graduate Program, University of California, La Jolla, San Diego, California 92093, United States; § Department of Neurosciences and Department of Pharmacology, School of Medicine, University of California, La Jolla, San Diego, California 92093, United States

## Abstract

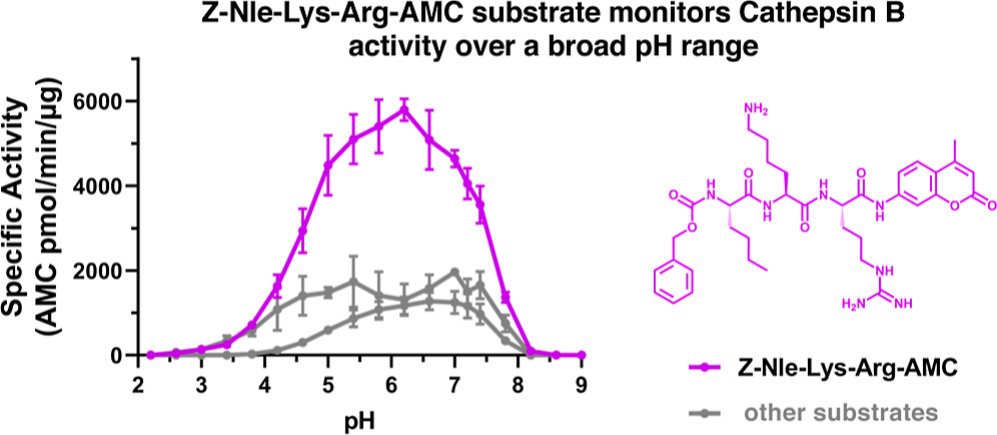

The biological and pathological functions of cathepsin B occur in acidic lysosomes and at the neutral pH of cytosol, nuclei, and extracellular locations. Importantly, cathepsin B displays different substrate cleavage properties at acidic pH compared to neutral pH conditions. It is, therefore, desirable to develop specific substrates for cathepsin B that measure its activity over broad pH ranges. Current substrates used to monitor cathepsin B activity consist of Z-Phe-Arg-AMC and Z-Arg-Arg-AMC, but they lack specificity since they are cleaved by other cysteine cathepsins. Furthermore, Z-Arg-Arg-AMC monitors cathepsin B activity at neutral pH and displays minimal activity at acidic pH. Therefore, the purpose of this study was to design and validate specific fluorogenic peptide substrates that can monitor cathepsin B activity over a broad pH range from acidic to neutral pH conditions. In-depth cleavage properties of cathepsin B were compared to those of the cysteine cathepsins K, L, S, V, and X via multiplex substrate profiling by mass spectrometry at pH 4.6 and pH 7.2. Analysis of the cleavage preferences predicted the tripeptide Z-Nle-Lys-Arg-AMC as a preferred substrate for cathepsin B. Significantly, Z-Nle-Lys-Arg-AMC displayed the advantageous properties of measuring high cathepsin B specific activity over acidic to neutral pHs and was specifically cleaved by cathepsin B over the other cysteine cathepsins. Z-Nle-Lys-Arg-AMC specifically monitored cathepsin B activity in neuronal and glial cells which were consistent with relative abundances of cathepsin B protein. These findings validate Z-Nle-Lys-Arg-AMC as a novel substrate that specifically monitors cathepsin B activity over a broad pH range.

## Introduction

Cathepsin B is a lysosomal cysteine protease that participates in protein degradation to maintain cellular balance of functional protein pathways.^1−3^ Cathepsin B belongs to the family of cysteine cathepsin proteases that together function in lysosomal protein homeostasis.^4,5^ Cathepsin B and other cysteine cathepsin family members normally function within lysosomes at acidic pH 4.6.^6−8^ However, in numerous human diseases of neurological disorders^9−20^ combined with infectious diseases, inflammation, and related conditions,^21−26^ cathepsin B leaks out of the lysosome into the cytosol of neutral pH 7.2.^27,28^ Cathepsin B retains its enzymatic activity at cytosolic neutral pH and activates cell death^29−32^ and inflammatory^33−36^ pathways that result in disease pathogenesis. In addition to the significant cellular function of cathepsin B at the neutral pH of the cytosol, cathepsin B is also present at neutral pH cellular locations of the nucleus^37,38^ as well as extracellular locations, especially in human diseases such as Alzheimer’s disease^39,40^ and cancer.^41−43^

The prominent biological functions of cathepsin B at distinct acidic and neutral pH environments of tissues^44−46^ indicate the need to specifically monitor cathepsin B activity over a broad pH range, without detecting other cysteine cathepsins. Currently, no such substrates exist because Z-Phe-Arg-AMC that is routinely used to assay cathepsin B in the field also monitors cathepsin L and other cysteine protease activities.^44,45,47−50^ Furthermore, Z-Arg-Arg-AMC has been found as a specific substrate for cathepsin B over other cysteine cathepsins,^44,48^ but this substrate preferentially monitors the neutral pH activity of cathepsin B rather than the enzyme’s acidic activity.^44^ Therefore, the purpose of this study was to rationally design selective peptide-AMC substrates that specifically monitor cathepsin B activity with high specific activity at both acidic and neutral pH conditions, without cleavage by other cysteine cathepsins.

The strategy of this study was to design specific substrates for cathepsin B based on its unique pH-dependent cleavage properties at acidic pH 4.6 compared to those at neutral pH 7.2. Cleavage properties of cathepsin B were determined by multiplex substrate profiling mass spectrometry (MSP-MS) that utilizes a defined peptide library designed to contain all known amino acids adjacent to protease cleavage sites.^51^ These substrate properties of cathepsin B were compared to those of other cysteine cathepsins L, K, S, V, and X at acidic and neutral pH conditions. The unique cleavage properties of cathepsin B allowed design of Z-peptide-AMC substrates for assessment of broad pH monitoring of cathepsin B activity, with specificity shown by a lack of cleavage by other cysteine cathepsins.

Results identified Z-Nle-Lys-Arg-AMC that monitors high specific activity of cathepsin B at acidic to neutral pHs, and this substrate is specific for cathepsin B over other cysteine cathepsins L, K, S, and V. Kinetic studies demonstrated greater catalytic efficiency of Z-Nle-Lys-Arg-AMC for analysis of acidic and neutral pH cathepsin B activity compared to the Z-Arg-Arg-AMC substrate, currently used in the field as a specific cathepsin B substrate.^44,48^ Furthermore, the novel Z-Nle-Lys-Arg-AMC substrate was demonstrated to specifically measure cathepsin B activity in neuronal and glial cells that contain varying levels of cathepsin B and other cathepsins (determined by proteomics assessment). Overall, the novel Z-Nle-Lys-Arg-AMC substrate advantageously monitors specific cathepsin B activity over a broad pH range of acidic to neutral pH conditions and, thus, will be useful for the assessment of cathepsin B activity at physiological pH conditions.

## Materials and Experimental Details

### Materials: Cathepsin B and Cysteine Cathepsins, Substrates, MSP-MS Library and Nano-LC–MS/MS, Cell Culture, and Proteomics

Cathepsin B and cysteine cathepsins (human, recombinant), were obtained from R&D Systems (Minneapolis, MN) or Abcam (Cambridge, MA). Proteases obtained for this study were procathepsin B (R&D Systems, #953-CY-010), active cathepsin L (R&D Systems, #952-CY-010), active cathepsin K (Abcam, #ab157067), procathepsin S (R&D Systems, #1183-CY-010), procathepsin V (R&D Systems, #1080-CY-010), and procathepsin X (R&D, #934-CY). Mouse cathepsin B was obtained from R&D Systems (R&D Systems, #965-CY-010). The UniProt identification numbers for the human cathepsin proteases studied are P07858 for cathepsin B, P07711 for cathepsin L, P43235 for cathepsin K, P25774 for cathepsin L, 060911 for cathepsin V, and Q9UBR2 for cathepsin X (also known as cathepsin Z). The Uniprot identification for mouse cathepsin B is P10605.

The substrates Z-KR-AMC, Z-GKR-AMC, Z-VKR-AMC, Z-YKR-AMC, Z-FKR-AMC, Z-WKR-AMC, Z-LKR-AMC, Z-AKR-AMC, Z-nRR-AMC, and Z-nKR-AMC (“n” represents norleucine) were custom-synthesized by GenScript (Piscataway, NJ). Z-RR-AMC was purchased from Bachem (#4004789) (Torrance, CA). Z-FR-AMC was purchased from Anaspec (#AS-24096) (Fremont, CA).

The design and synthesis of the 228 14-mer peptide library used for MSP-MS assays are described in ref (51). MSP-MS assays utilized low-binding 600 μL microtubes (Corning, Reynosa, MX), dithiothreitol (DTT) (#V351, Promega, Madison, WI), urea (#U2222, Teknova, Hollister, CA), HPLC-grade water (#W6-4, Fisher Scientific), citric acid monohydrate (#1.00244.0500, Merck, Burlington, MA), sodium phosphate dibasic anhydrous (#SX-072305, EMD, Burlington, MA), sodium acetate (#BP-333-500, Fisher Scientific, Fair Lawn, NJ), EDTA (#324503, Calbiochem, Burlington, MA), sodium chloride (#S271-1, Fisher Chemical, Pittsburgh, PA), acetonitrile (#A955-4, Fisher Chemical, Pittsburgh, PA), formic acid (FA) (#A117-50, Fisher Chemical, Pittsburgh, PA), trifluoroacetic acid (TFA) (#A116-50, Fisher Chemical, Pittsburgh, PA), C18 LTS Tips (#PT-LC18-960, Rainin, Oakland, CA), C18 for SPE stage-tips (#2215-C18, 3M Co., Maplewood, MN), and BEH C18 packing material (#186004661, Waters Corp., Milford, MA).

Cell culture of human neuroblastoma (SHSY-5Y and SK-N-MC) and mouse microglia (BV2) utilized reagents consisting of RPMI 1640, MEMa, F-12K, heat-inactivated fetal bovine serum, and phosphate-buffered saline (PBS) (catalog numbers: 11875093, 12561056, 30-2004, and 10082147, 10010023, respectively, from Gibco, Grand Island, NY). For proteomics, cells were homogenized in a buffer containing a cocktail of protease inhibitors of pepstatin A, leupeptin, chymostatin, and AEBSF (catalog numbers: 516481, 108976, 230790, and 101500, respectively, from Millipore Burlington, MA), and E64c (catalog number: N-1655 from Bachem, Torrance, CA).

Proteomics of cells utilized trypsin/Lys-C (V5037, Promega Madison WI), Empore C18 (octadecyl) (2215, from 3M, St, Paul, MN), and peptide assay (23,275, Pierce Waltham, MA) for sample preparation, combined with nano-LC–MS/MS reagents of trifluoroacetic acid, formic acid, Optima LC–MS water, Optima LC–MS acetonitrile (A116, 85178, W65, and A955, respectively, from Fisher Scientific, Waltham, MA). LC columns utilized were a pricofrit self-pack column of 360 μm OD, 75 μm ID, and 15 μm tip (PF360-75-15-N from New Objective, Littleton, MA) and an Acquity UPLC BEH C18 (130 Å, 1.7 μm) (186004661, from Waters Corp. Milford, MA).

### Activation of Cathepsin B and Cysteine Cathepsin Proteases

Recombinant human procathepsins B, V, and S were activated by incubation at 37 °C for 30 min in the 20 mM Na-acetate pH 5.5, 100 mM NaCl, 5 mM DTT, 1 mM EDTA activation buffer. Recombinant human procathepsin X was activated by incubation at room temperature for 5 min in the 20 mM citrate phosphate pH 3.5, 100 mM NaCl, and 5 mM DTT activation buffer.

### Cathepsin B Activity with Z-Arg-Arg-AMC and Z-Phe-Arg-AMC Substrates at pH 4.6 and 7.2

Cathepsin B (0.04 ng/μL) activity was assessed with Z-Arg-Arg-AMC and Z-Phe-Arg-AMC substrates (40 μM final concentration) in 40 mM citrate phosphate buffer pH 4.6 or Tris–HCl pH 7.2 in 1 mM EDTA, 100 mM NaCl, and 5 mM DTT with incubation at room temperature (25 °C) in triplicate, and AMC fluorescence readings per second (RFU/s) (excitation 360 nm, emission 460 nm) were recorded over a period of 30 min by a BioTek Synergy HTX plate reader (Software version 3.08.01). Enzyme initial velocity was calculated using the highest slope recorded for 10 consecutive fluorescent readings within the initial 30 min. The mean and standard deviation (SD) were determined from triplicates. RFU/s were converted to pmol/min/μg (specific activity) using AMC standard curves. All data were plotted, calculated, and analyzed using GraphPad Prism (version 9.4.1).

**Table 1:** Z-Phe-Arg-AMC and Z-Arg-Arg-AMC Substrates of Cathepsin B Are Cleaved by Several Cysteine Cathepsins^a^

protease	substrate			
	Z-Phe-Arg-AMC		Z-Arg-Arg-AMC	
	pH 4.6	pH 7.2	pH 4.6	pH 7.2
cathepsin B	974	895	130	441
cathepsin L	7099	0	125	0
cathepsin K	332	420	0	0
cathepsin S	21	29	0	0
cathepsin V	546	0	23	0

a The specific activity for cathepsins B, L, K, S, and V were determined for each substrate Z-Phe-Arg-AMC and Z-Arg-Arg-AMC (40 μM) at pH 4.6 and pH 7.2 as described in the Materials and Experimental Details.

### Protease Substrate Cleavage Profiling by MSP-MS

#### Peptide Library for Substrate Profiling by MS

MSP-MS was performed for cathepsins B, L, K, S, and V at pH 4.6 and 7.2 by methods that we have described previously.^51^ Cathepsin X was performed only at pH 4.6. In a total volume of 22 μL, cathepsin B (0.1 ng/μL), cathepsin L (0.04 ng/μL), cathepsin K (0.07 ng/μL), cathepsin S (1.2 ng/μL), cathepsin V (0.16 ng/μL), and cathepsin X (0.09 ng/μL) were incubated with a mixture of 228 14-mer peptides (0.5 μM for each peptide) in 50 mM citrate phosphate at pH 4.6 or pH 7.2, 100 mM NaCl, 5 mM DTT, 1 mM EDTA assay buffer for 15 and 60 min at 25 °C. 10 μL was removed at 15 and 60 min to be combined with 60 μL of 8 M urea. A control assay used inactivated cathepsins by incubating it with 8 M urea for 60 min at 25 °C, prior to addition of the peptide library in the assay buffer. Assays were conducted in quadruplicate. Samples were acidified by addition of 40 μL of 2% TFA and desalted using custom-made C18 spin tips. The collected liquid samples were dried completely in a vacuum centrifuge and then stored at −70 °C. For LC–MS/MS analysis, dried samples were resuspended in 40 μL of 0.1% TFA, and 4 μL was injected into the Thermo Fisher Scientific Q-Exactive mass spectrometer with an Ultimate 3000 HPLC. Peptides were separated by reverse-phase chromatography using a C18 column at (65 °C), which was done as we have previously described.^50,51^

#### PEAKS Bioinformatics Analysis

PEAKS (v 8.5) software (Bioinformatics Solutions Inc.) was used for MS/MS data analysis. The 228 14-mer library sequence was used as the database for MS data searching and matching, and as a control it was also searched with a decoy consisting of the 228 14-mer library sequences in a reverse order. A precursor tolerance of 0.01 Da and 20 ppm for MS2 fragments was used. Data were filtered to 0.9% peptide sequence false discovery rates determined from hits from decoys. Label-free quantification (LFQ) was used for peptide quantification, and the data was normalized by LOESS-G normalization method using Normalyzer tool version 1.1.1.^52^ Outliers from groups of replicates were removed by Dixon’s Q test.^53^ Missing and zero values were treated the same and replaced with imputed values determined from randomized normally distributed numbers within the range of the smallest 5% dataset ± SD. The control 0 min values in MSP-MS obtained for cathepsins B, L, K, S, V, and X at pH 4.6 were also analyzed by PEAKS (for *n* = 20). To determine if the peptide intensity data in at least one of the groups of the 0, 15, and 60 min timepoints were statistically significant, ANOVA testing with multiple testing correction was used; those with *q* < 0.05 were considered for further analysis. Cleaved peptide products at the 15 or 60 min timepoints were defined as having intensity scores of 8-fold or more with *p* < 0.05 by the two-tailed homoscedastic *t*-test.

#### Cleavage Site Analysis by iceLogo

Evaluation of the frequencies of the P4 to P4′ amino acids adjacent to the cleavage sites was conducted using the iceLogo software 1.3.8, where the “experimental data set” consists of the detected cleavage sites defined earlier and the “reference data set” that consists of all possible cleavages within the MSP-MS library of 228 14-mer peptides. *z*-scores were calculated by the equation (*X* – μ)/σ, where *X* is the frequency of the amino acid occurring in the “experimental data set” at a specific position relative to the cleavage site (e.g., P3, P2, and P1), μ is the frequency of the amino acid at a specific position in the “reference data set”, and σ is the SD. *z*-scores were utilized to generate iceLogo illustrations of the relative frequencies of amino acid residues at each of the P3 to P1 positions of the cleaved peptides where heights of the single letter amino acids represent “percent difference”, defined as the difference in the frequency for an amino acid appearing in the “experimental data set” relative to the “reference data set”. Amino acids shown above the midline have positive *z*-scores, indicating preferred amino acids, and amino acids shown below the midline have negative *z*-scores, indicating not preferred amino acids, illustrated using *p* < 0.30 cutoff criteria in the iceLogo software with *p* < 0.05 labeled in purple.

### Analysis of pH-Dependent Cathepsin B Activity with Fluorogenic Substrates Designed from MSP-MS Data

For comparison of cathepsin B activity at pH 4.6, pH 5.5, and pH 7.2 with multiple fluorogenic substrates, cathepsin B (0.04 ng/μL) was incubated with 40 μM substrates consisting of Z-Lys-Arg-AMC, Z-Phe-Arg-AMC, Z-Arg-Arg-AMC, Z-Gly-Lys-Arg-AMC, Z-Val-Lys-Arg-AMC, Z-Tyr-Lys-Arg-AMC, Z-Phe-Lys-Arg-AMC, Z-Trp-Lys-Arg-AMC, Z-Leu-Lys-Arg-AMC, Z-Ala-Lys-Arg-AMC, Z-Nle-Arg-Arg-AMC, and Z-Nle-Lys-Arg-AMC. AMC product formation (RFU/s) monitored in 40 mM citrate phosphate, pH 4.6 and pH 7.2, 1 mM EDTA, 100 mM NaCl, and 5 mM DTT was determined from the average of RFU/s triplicates at each pH condition.

For analysis of pH-dependent activity of cathepsin B (0.04 ng/μL), proteolytic activity was monitored over the pH range of pH 2.2–9.0 in 40 mM citrate phosphate (pH 2.2 to pH 6.6) and 40 mM Tris–HCl (pH 7.0 to 9.0), 1 mM EDTA, 100 mM NaCl, and 5 mM DTT, with preincubation in each pH buffer for 10 min prior to initiating the assay by addition of Z-Nle-Lys-Arg-AMC, Z-Arg-Arg-AMC, or Z-Phe-Arg-AMC to a final concentration of 40 μM to generate the pH curve data.

For evaluation of cathepsin B and cysteine cathepsins for substrate selectivity, each cathepsin was incubated with 40 μM substrates consisting of Z-Phe-Arg-AMC, Z-Arg-Arg-AMC, and Z-Nle-Lys-Arg-AMC, and the activity was monitored in 40 mM citrate phosphate (pH 4.6, pH 5.5 and pH 7.2), 1 mM EDTA, 100 mM NaCl, and 5 mM DTT for cathepsin B (0.04 ng/μL), cathepsin K (0.03 ng/μL), cathepsin L (0.03 ng/μL), cathepsin S (0.14 ng/μL), cathepsin V (0.04 ng/μL), and cathepsin X (0.20 ng/μL). Since cathepsin X cannot cleave any of the AMC substrates, MCA-RPPGFSAFK(Dnp)-OH (R&D Systems #ES005) was used to verify that cathepsin X was active.

### Kinetic Parameters of Cathepsin B Assessed by *k*_cat_ and *K*_m_ Values with Fluorogenic Substrates

The kinetic parameters of *k*_cat_ and *K*_m_ for Z-Nle-Lys-Arg-AMC, Z-Arg-Arg-AMC, and Z-Phe-Arg-AMC substrates were determined at pH 4.6 and pH 7.2, using substrate concentrations of 225–5.9 μM with 0.04 ng/μL cathepsin B. RFU values were converted to s^–1^ using AMC standard curves. The *k*_cat_ and *K*_m_ values were obtained from curve fitting the converted RFU data using GraphPad Prism9 software to the equation *v*_0_ = (*V*_max_[S])/(*K*_m_ + [S]), where *v*_0_ is the initial velocity of the enzyme with its corresponding [S] substrate concentration and *V*_max_ is the maximum enzyme velocity at saturated [S]. *V*_max_ = *k*_cat_[E]_T_, where [E]_T_ is the total cathepsin B concentration used in the assay. *K*_m_ is the *x*-axis value [S], where *y* = *V*_max_/2. SD values for *k*_cat_ and *K*_m_ were determined from curving fitting the *v*_0_ and [S] data from triplicates.

### Neuronal and Microglial Cell Culture

Human neuroblastoma SH-SY5Y^54^ and SK-N-MC^55^ cells and embryonic mouse microglia BV2 (gifted by Christopher Glass, Ph.D, UC San Diego) were grown in 5% CO_2_ at 37 °C under sterile conditions. Growth media conditions were 10% heat-inactivated fetal bovine serum (Life Technologies, Carlsbad, CA) and media consisting of 90% MEMa (Life Technologies) for SK-N-MC cells, 45% Ham’s F12-K (ATCC, Manassas, VA), and 45% MEMa (Life Technologies) for SH-SY5Y and 90% RPMI (Life Technologies) for BV2.

### Cathepsin B Activity in Human Neuroblastoma and Mouse Microglia

Characterization of the Z-Nle-Lys-Arg-AMC substrate in proteolytic activity assays for specific cathepsin B activity of cell homogenates (prepared in 0.32 M sucrose) was conducted at 40 mM citrate phosphate pH 4.6, 5.5, and 7.2, 5 mM DTT, 1 mM EDTA, 100 mM NaCl, 1.2% DMSO, and homogenates of human neuroblastoma cell lines, SHSY-5Y and SK-N-MC, and mouse microglia cell line, BV2. A concentration of 60 μM Z-Nle-Lys-Arg-AMC, Z-Arg-Arg-AMC, and Z-Phe-Arg-AMC substrates was used in the proteolytic activity assays. Cell homogenates were diluted 1:10 prior to addition to assays. Homogenate protein concentrations were measured by the Biorad protein assay kit (Biorad #5000113, Hercules, CA). The cathepsin B specific inhibitor, CA-074, was used to specifically inhibit cathepsin B activity at concentrations of 1 μM at pH 4.6 and 5.5 and 10 μM at pH 7.2. Assays were performed in 96-well plates at room temperature (25 °C) in a total volume of 100 μL with triplicates. Cell homogenates were incubated with CA-074 or inhibitor controls at RT for 30 min in the absence of substrate, followed by addition of the substrate and incubation at 37 °C. Fluorescence was quantified at 15, 30, and 60 min after substrate was added, by a Biotek Synergy HTX microplate reader with excitation at 360 nm, emission at 460 nm, gain 50, top optics, and read height at 1 mm. Prism GraphPad software was used to analyze data.

### Proteomics Identification and Quantitation of Cysteine Cathepsins in Neuronal and Microglial Cells

Analysis of neuronal and microglial cells by proteomics MS utilized protocols that we have previously described elsewhere.^56,57^ To summarize, approximately 10^8^ cells were collected from 90% confluent flasks with PBS washing and centrifugation at 500*g*. Cell pellets were lysed by dounce homogenization in ice-cold 100 mM Tris pH 7.4, 50 mM NaCl, 1 mM EDTA, and protease inhibitors [10 μM pepstatin A, 10 μM leupeptin, 10 μM chymostatin, 100 μM AEBSF (Millipore, Burlington, MA), and 10 μM E64c (Bachem, Torrance, KA)]. Tryptic digests for proteomics analyses were prepared from 200 mg of total cellular protein as described previously,^59^ with two biological flask replicates per cell line and triplicate technical replicate injections per biological replicate. Tryptic peptides were diluted to 0.5 mg/mL in 2% acetonitrile, 0.1% trifluoroacetic acid, and 2 μg total amount injected by nano-LC–MS/MS on a Dionex UltiMate 3000 nano LC and Q-Exactive mass spectrometer (Thermo Fisher Scientific). Spectra were acquired as described previously.^59^ For protein identification and quantitation, spectra were queried against a custom database consisting of the protein sequences of 15 human cathepsins (SH-SY5Y and SK-N-MC) or 15 mouse cathepsins (BV2) with PEAKS v. 8.5 using decoy-fusion and LFQ methods (Bioinformatics Solutions Inc., Waterloo, ON), with search parameters described previously.^59^ Proteins were considered identified if present in at least 2 of 3 technical replicates and at least 1 biological replicate. Proteins were considered quantifiable based on the same criteria as for identification. Peak intensity areas of quantifiable data are expressed as mean ± SD.

## Results

### Analysis of Cathepsin B Substrates Z-Phe-Arg-AMC and Z-Arg-Arg-AMC for Cleavage by Cysteine Cathepsins Demonstrate Lack of Specificity

We compared the specific activity of human cathepsin B with the fluorogenic substrates Z-Phe-Arg-AMC and Z-Arg-Arg-AMC with that of other cysteine cathepsins (Table 1). Both of these substrates were cleaved by cathepsin B and other cysteine cathepsins L, K, S, and V, indicating that these substrates are not specific for cathepsin B. These assays also demonstrated that cathepsins B, K, and S were active at both acidic and neutral pHs of 4.6 and 7.2, respectively, but cathepsins L and V displayed activity only at acidic pH 4.6 and not at pH 7.2. These data are consistent with others with respect to the pH properties of these cathepsins.^44,45,47−50^ Significantly, these findings confirm the lack of specificity of Z-Phe-Arg-AMC and Z-Arg-Arg-AMC substrates for cathepsin B.

Comparison of Z-Arg-Arg-AMC cleavage by cathepsin B at acidic lysosomal pH 4.6 and at neutral pH 7.2 of the cytosol showed that this substrate displays pH-dependent cleavage properties. Z-Arg-Arg-AMC is readily cleaved by cathepsin B at neutral pH, but this substrate has poor cleavage activity at acidic pH that is one-third of the specific activity at neutral pH.

The lack of specificity of Z-Phe-Arg-AMC and Z-Arg-Arg-AMC for cathepsin B compared to other cysteine cathepsins, and preference of Z-Arg-Arg-AMC to monitor neutral pH cathepsin B, indicates the need for the development of specific substrates of cathepsin B operating over a broad pH range.

### Cathepsin B Displays Distinct Cleavage Properties Compared to Other Cysteine Cathepsins Revealed by MSP-MS

The substrate cleavage properties of human cathepsin B were compared to that of the cysteine cathepsins K, L, V, S, and X by MSP-MS analysis. MSP-MS was conducted by incubation of each protease with a library of 228 14-mer peptides at acidic pH 4.6 and neutral pH 7.2, and cleavage products were identified and analyzed using LC–MS/MS. The peptide library used for MSP-MS analysis was designed to contain all known protease cleavage sites with respect to amino acid residues at cleavage sites combined with neighboring residues.^51^ These cysteine proteases cleaved peptides at both pH 4.6 and pH 7.2 in the MSP-MS study, with the exception of cathepsin X that generated a low number of cleavage products at only pH 4.6 (shown by volcano plots in Supporting Information Figure S1).

**Figure 1:**
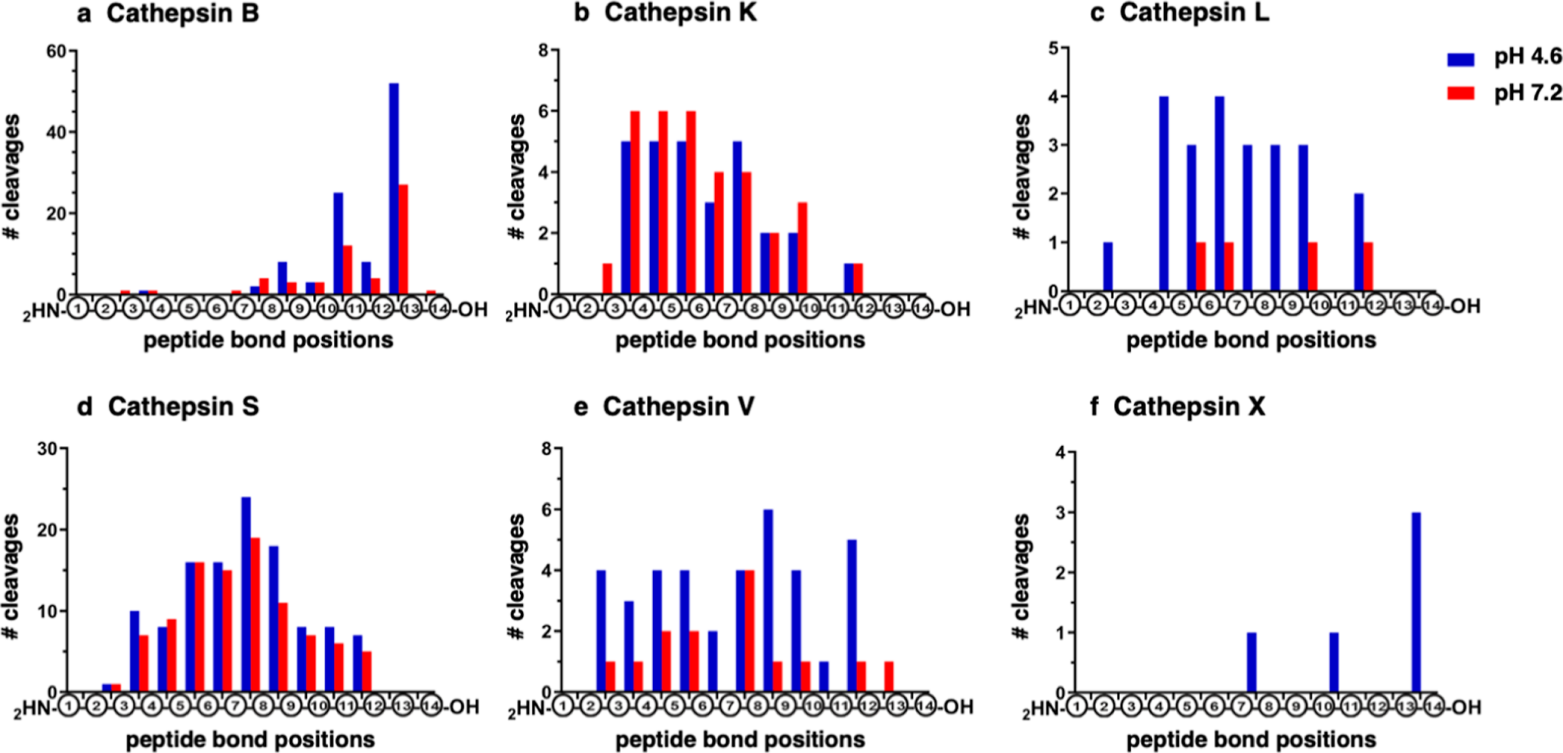
Exopeptidase and endopeptidase cleavages by cathepsin B compared to other cysteine cathepsins assessed by MSP-MS. The proteolytic cleavage properties of cathepsin B and cysteine cathepsins K, L, S, V, and X were assessed at pH 4.6 and pH 7.2 by substrate profiling with the peptide library in MSP-MS assays. MSP-MS analyses were conducted for cathepsins B, K, L, S, V, and X as shown in panels (a–f), respectively. After incubation of each protease with the peptide library for 15 min, with the exception that cathepsin X was incubated with the library for 60 min, cleavage products were identified by nano-LC–MS/MS as described in the methods. The number of cleavages at each peptide bond, numbers 1 to 13, is illustrated for the cysteine cathepsins B, K, L, S, V, and X.

MSP-MS analysis of cleavage site locations within the 14-mer peptides showed that cathepsin B differs in its exopeptidase and endopeptidase cleavages compared to the other cysteine cathepsins. Cathepsin B displayed major dipeptidyl carboxypeptidase (DPCP) cleavages occurring between residues 12 and 13 of the peptide substrates, as well as lower endopeptidase cleavages occurring within the 14-mer substrates (Figure 1a). The dual DPCP and endopeptidase data of cathepsin B activity is consistent with other studies.^4,5,44^ These DPCP and endopeptidase activities of cathepsin B distinguish it from the cathepsins K, L, S, and V, which displayed endopeptidase activities and no carboxypeptidase activity (Figure 1b–e). Cathepsin X displayed endopeptidase and monopeptidyl carboxypeptidase cleavage (Figure 1f) (with a low number of five peptides cleaved), which differ from that of cathepsin B.

The substrate cleavage specificity of cathepsin B was compared to that of the other cysteine cathepsins by assessing preferred residues at the P4–P4′ positions surrounding the P1–P1′ cleavage site using *z*-scores and iceLogo (Supporting Information Figures S2 and S3). Heat maps of *z*-scores illustrated the preferred residues (green shade) and non-preferred residues (yellow shade) at each of the P4 to P4′ positions. All amino acids were assessed with the exception of methionine and cysteine; norleucine (n) was used as a sulfur-free isostere of methionine.

**Figure 2:**
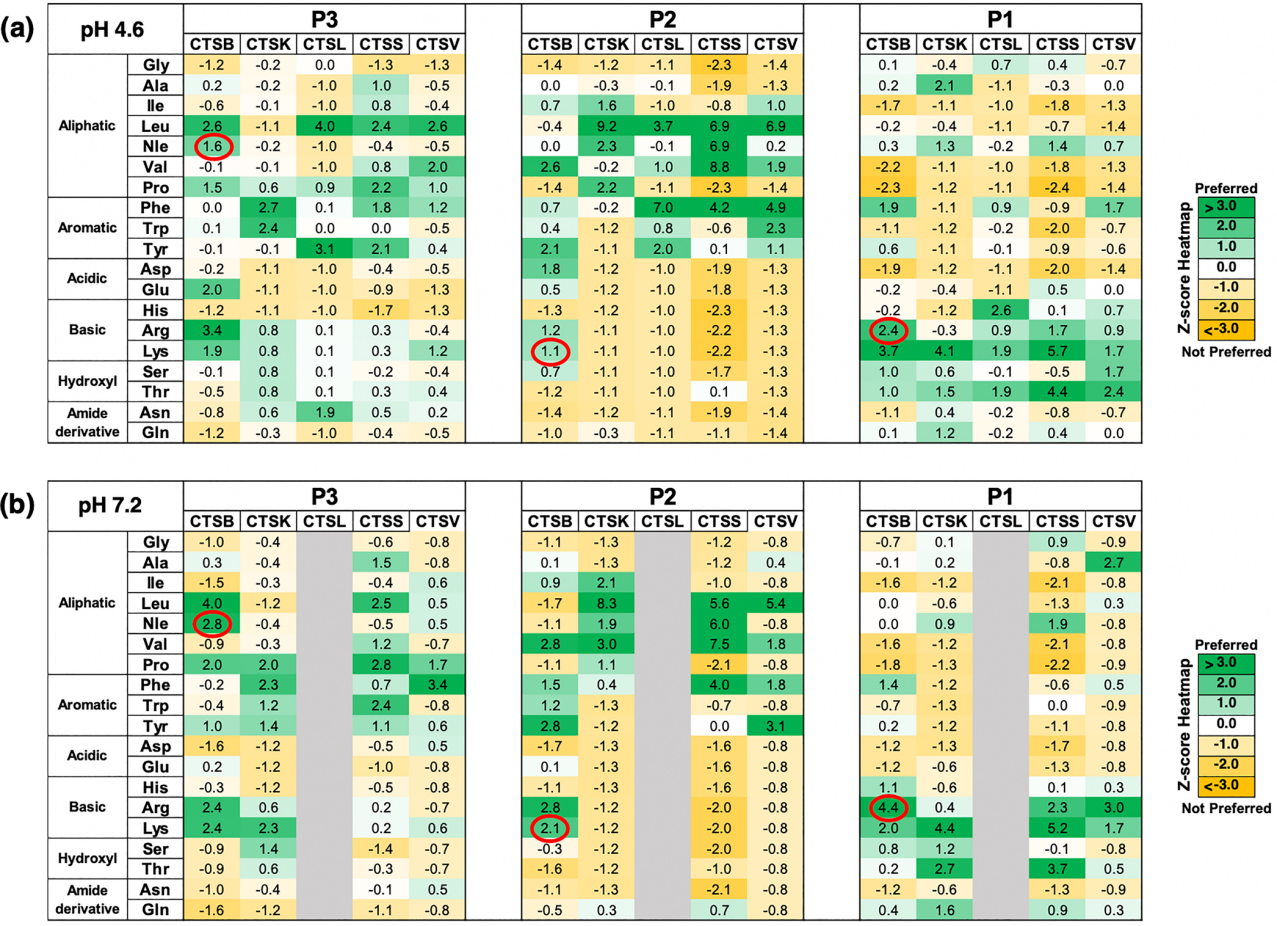
Assessment of distinct cathepsin B preferred residues at P1 to P3 positions adjacent to P1–P1′ cleavage sites compared to other cysteine cathepsins. The preferred residues of human cathepsin B at P1 to P3 positions were compared to that of cathepsins K, L, S, and V at pH 4.6 (panel a) and pH 7.2 (panel b) shown in heat maps of *z*-scores. Preferred residues are indicated by positive scores (green shades), and non-preferred residues are indicated by negative scores (yellow shades). The residues circled in red indicate unique residues preferred by cathepsin B over the other cathepsins at the P1 to P3 positions. The *z*-scores represent comparisons of the frequencies of amino acids at each of these positions (relative to the cleavage sites) of our MSP-MS experimental with the “reference data set” that covers all 2964 possible cleavage sites of the peptide library. The *z*-scores were, thus, calculated using the equation (*X* – μ)/σ, where *X* represents the frequency of a specific amino acid in the “experimental data set”, μ denotes the frequency of that amino acid at a particular position in the “reference data set”, and σ signifies the standard deviation. Positive *z*-scores indicate preferred residues and negative scores indicate not-preferred residues, shown in the heat map as green to yellow shading for high to low *z*-scores.

Comparison of the heat maps at pH 4.6 and pH 7.2 for cathepsins B, K, S, and V showed differing patterns for each of these proteases at the acidic and neutral pH conditions (Supporting Information Figures S2 and S3). We focused on the preferred residues at positions P3 to P1 for cathepsin B (Figure 2) that can be utilized for designing specific Z-peptide-AMC fluorogenic substrates spanning acidic and neutral pH activities of cathepsin B.

For cathepsin B, we sought to identify amino acids that were preferred by cathepsin B at both pH 4.6 and pH 7.2 (Figure 2). At P3, P2, and P1, the enzyme displayed preferences for basic residues (Arg and Lys). In addition, norleucine (Nle) and Leu were preferred at P3, and Val was preferred at P2. Cathepsins K, S, L, and V had different P3 and P2 preferences compared to cathepsin B. In particular, Nle at the P3 position, and Arg and Lys at P2 were not preferred by cathepsins K, S, L, and V (Figure 2). Val at P2 was preferred by cathepsin K, S, L, and V at one or both pH conditions and, therefore, was not selective for cathepsin B. At the P1 position, cathepsins K, L, and S preferred Lys over Arg, while cathepsin V preferred Lys over Arg at pH 4.6 and the opposite at pH 7.2. Compilation of the preferred P3, P2, and P1 residues for the five enzymes revealed the distinct preferences of cathepsin B for Nle at the P3 position, Lys or Arg at P2, and Arg at P1. These cleavage properties suggested that a fluorogenic peptide consisting of tripeptides Z-Nle-Lys-Arg-AMC or Z-Nle-Arg-Arg-AMC would be selectively cleaved by cathepsin B at both pH 4.6 and pH 7.2 relative to the other four cysteine cathepsin enzymes.

### Design and Validation of Z-Nle-Arg-Lys-AMC as a Specific Cathepsin B Substrate for Acidic and Neutral pH Conditions

**Figure 3:**
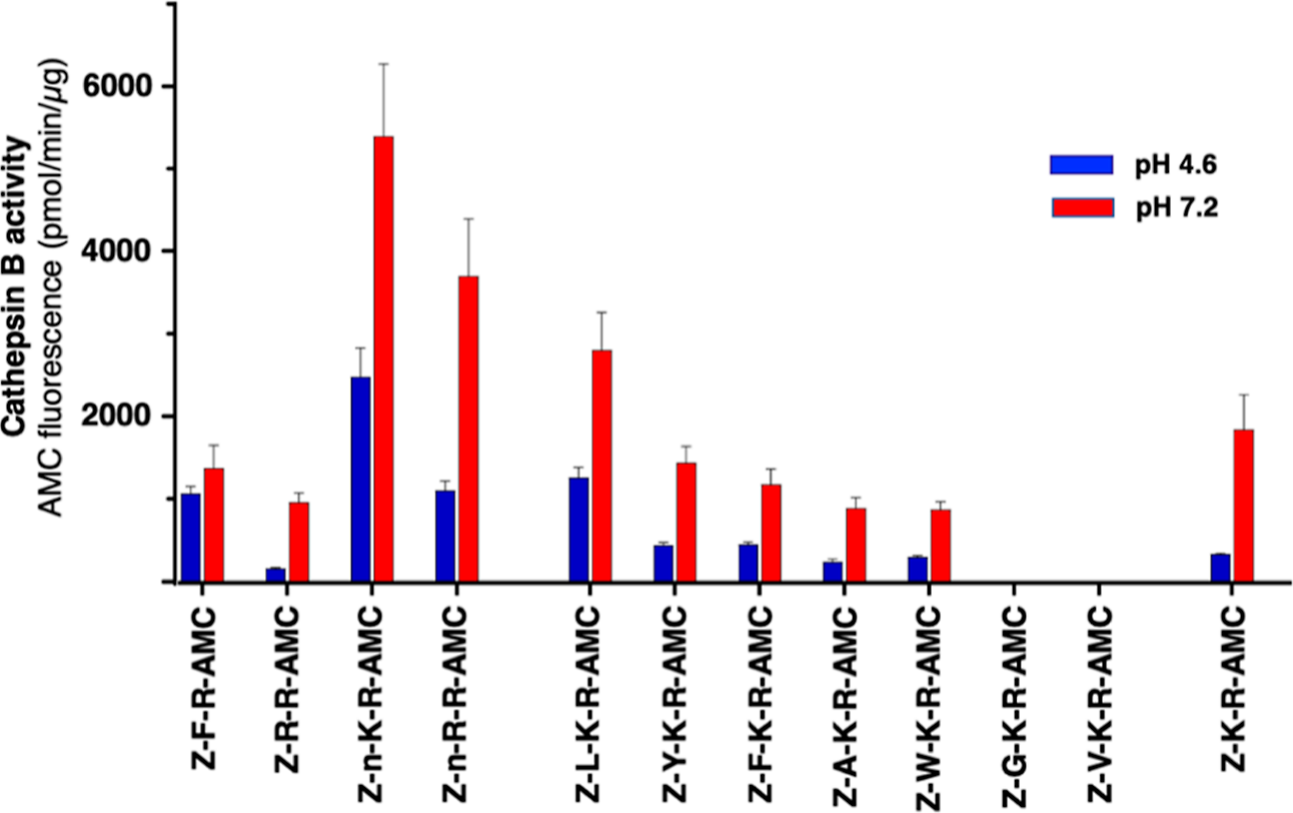
Z-Nle-Lys-Arg-AMC monitors high cathepsin B specific activity at pH 4.6 and pH 7.2 compared to related tripeptide and dipeptide substrates. Z-Nle-Lys-Arg-AMC (Z-n-K-R-AMC) and the indicated tripeptide and dipeptide fluorogenic substrates (at 40 μM) were compared for proteolysis by cathepsin B at pH 4.6 (blue bars) and pH 7.2 (red bars). One-letter codes for amino acids are shown for the peptidic substrates. Proteolytic activity of cathepsin B is shown as the mean ± SD (*n* = 4).

We synthesized Z-Nle-Lys-Arg-AMC and Z-Nle-Arg-Arg-AMC and quantified cathepsin B activity at acidic and neutral pH conditions (Figure 3). The specific activity of cathepsin B for cleaving Z-Nle-Lys-Arg-AMC was higher than all other substrates at both pHs. The Z-Nle-Arg-Arg-AMC substrate displayed about 30% and 50% less activity at pH 4.6 and pH 7.2, respectively, compared to Z-Nle-Lys-Arg-AMC. The Z-Nle-Lys-Arg-AMC substrate monitored higher cathepsin B specific activity compared to the commonly used cathepsin B substrates of Z-Phe-Arg-AMC and Z-Arg-Arg-AMC.

Modifications of the P3 residues of variant tripeptide sequences of Z-Nle-Lys-Arg-AMC were assessed (Figure 3). Substitution of Leu for Nle at the P3 position generated the substrate Z-Leu-Lys-Arg-AMC, which showed less activity than Z-Nle-Lys-Arg-AMC. When the P3 residue was modified to Tyr, Phe, Ala, or Trp, the resultant substrates displayed decreased activities at both acid and neutral pHs. When the P3 residue was changed to Gly or Val, no cathepsin B activity was observed, correlating with Gly and Val having negative *z*-scores in the MSP-MS data.

Assessment of the dibasic substrate of Z-Lys-Arg-AMC that lacks a P3 amino acid but has the most favorable P2 and P1 residues had lower activity than Z-Nle-Lys-Arg-AMC but higher activity than substrates that have an unfavorable amino acid at the P3 position.

Overall, analysis of Z-Nle-Lys-Arg-AMC and related peptide-AMC substrates demonstrated that the optimized residues at P3, P2, and P1 positions provided design and validation of the high specific activity of the novel Z-Nle-Lys-Arg-AMC substrate of cathepsin B.

### Z-Nle-Lys-Arg-AMC Substrate Monitors Cathepsin B Activity over a Broad pH Range

**Figure 4:**
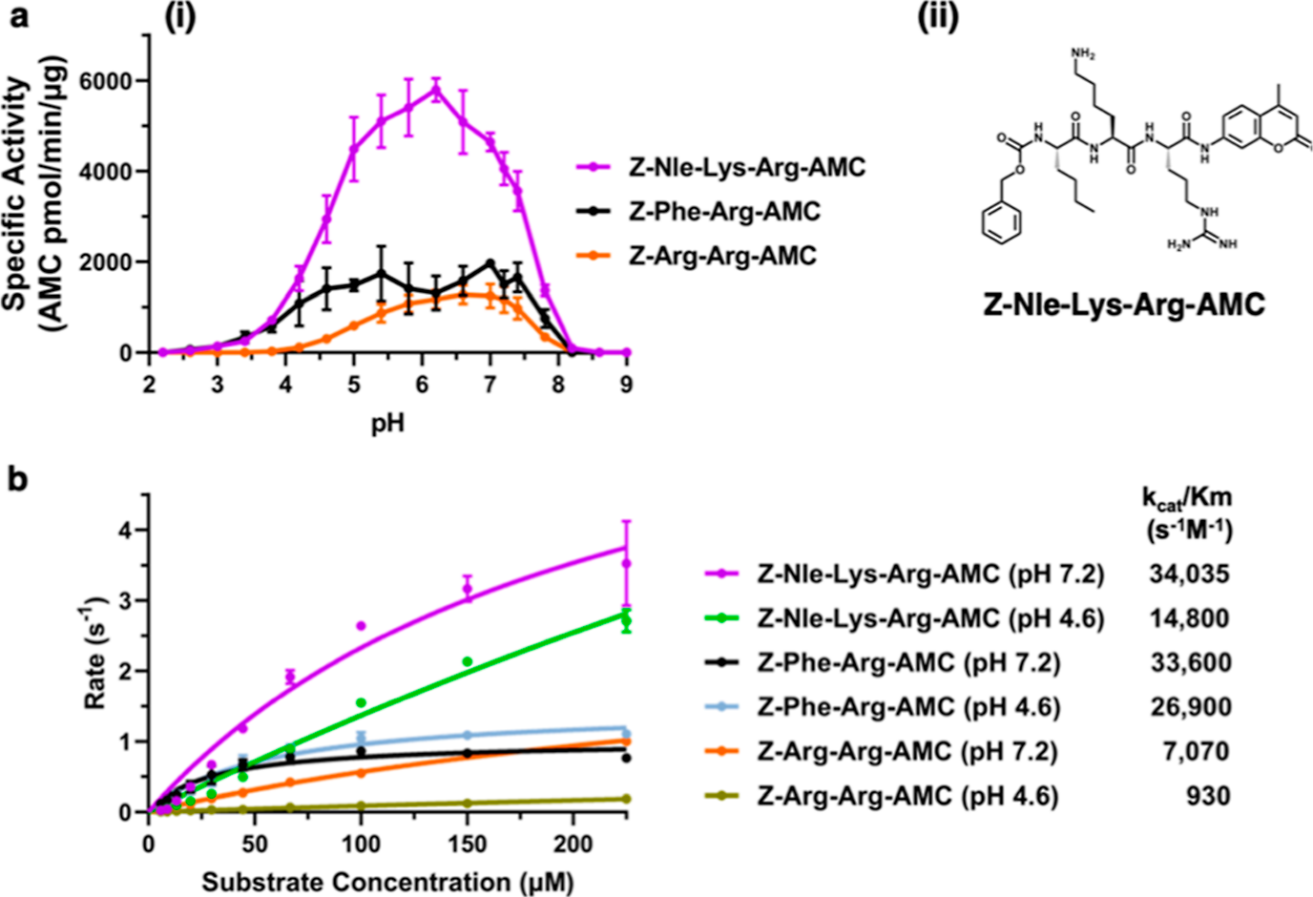
Cathepsin B cleaves Z-Nle-Lys-Arg-AMC over a broad pH range. (a) pH profile of human cathepsin B activity with Z-Nle-Lys-Arg-AMC compared to Z-Arg-Arg-AMC and Z-Phe-Arg-AMC substrates. (i) Cathepsin B pH profiles with three different substrates (40 μM) were assessed at pH 2 to 9. Data points are shown as the mean ± SD (*n* = 3). (ii) Structure of Z-Nle-Lys-Arg-AMC is shown. This substrate provides the highest specific activity for cathepsin B activity. (b) Kinetic *k*_cat_/*K*_m_ values for cathepsin B activity with the substrates Z-Nle-Lys-Arg-AMC, Z-Arg-Arg-AMC, and Z-Phe-Arg-AMC. *k*_cat_/*K*_m_ values for each of the substrates at pH 4.6 and pH 7.2 were calculated for cathepsin B by plotting the rate of AMC product formation of the *y*-axis with its respective substrate concentration of the *x*-axis as described in the methods.

The pH properties of Z-Nle-Lys-Arg-AMC were assessed and compared to the established substrates Z-Arg-Arg-AMC and Z-Phe-Arg-AMC (Figure 4). Z-Nle-Lys-Arg-AMC showed a broad optimum pH range of pH 4.5 to pH 7.5 that represented 50% of the maximal activity observed at pH 5.5–6.5, which was above the activity measurements monitored by the other two Z-Arg-Arg-AMC and Z-Phe-Arg-AMC substrates. The structure of Z-Nle-Lys-Arg-AMC illustrates its side chains of the tripeptide residues of this high activity substrate (Figure 4a). Kinetic assessment by *k*_cat_/*K*_m_ values showed the high catalytic efficiency of cathepsin B activity monitored with Z-Nle-Lys-Arg-AMC and lower catalytic efficiency with Z-Arg-Arg-AMC at both pH 7.2 and pH 4.6 (Figure 4b and Supporting Information Table S1). Z-Phe-Arg-AMC displayed high *k*_cat_/*K*_m_ values similar to Z-Nle-Lys-Arg at pH 7.2 and had higher catalytic efficiency at pH 4.6.

### Z-Nle-Lys-Arg-AMC Specifically Monitors Cathepsin B Activity Compared to Other Cysteine Cathepsins

**Figure 5:**
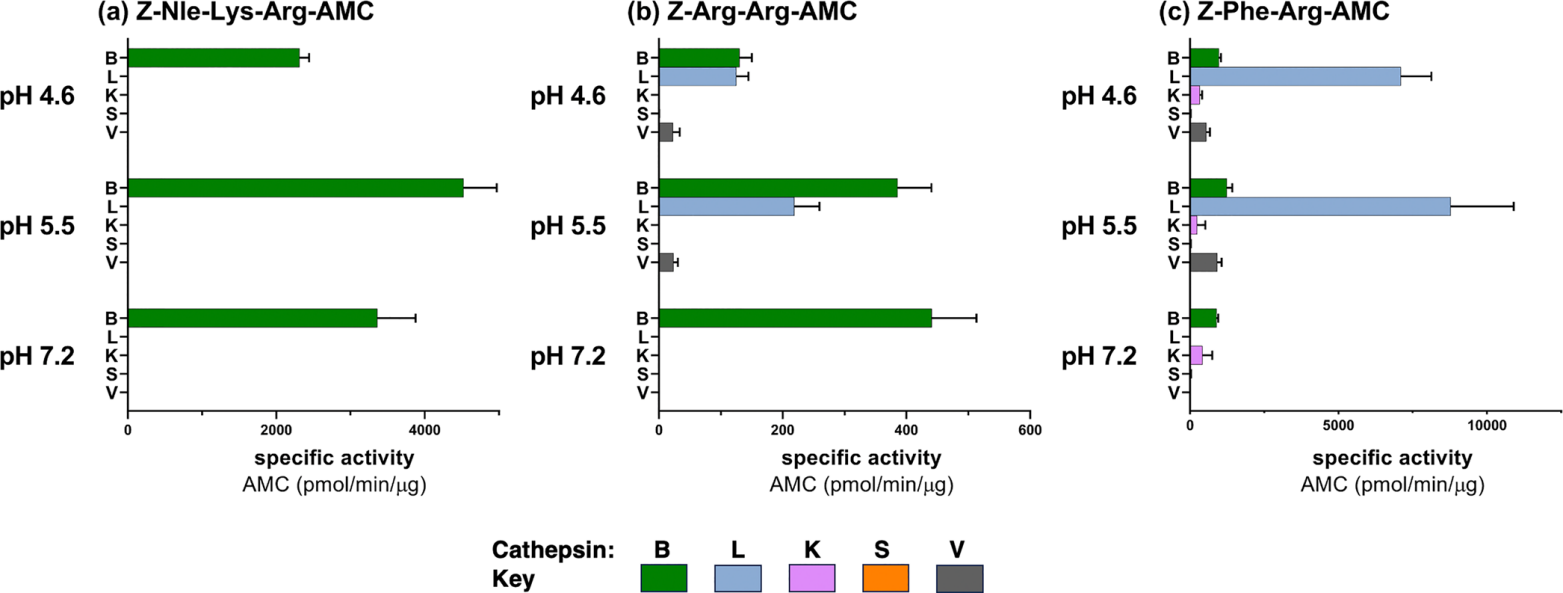
Z-Nle-Lys-Arg-AMC displays specificity for cathepsin B over other cysteine cathepsins L, K, S, and V. (a) Z-Nle-Lys-Arg-AMC: cathepsin B specific activity for cleavage of Z-Nle-Lys-Arg-AMC was compared to that of cathepsins L, K, S, and V at pHs 7.2, 5.5, and 4.6. (b) Z-Arg-Arg-AMC: cathepsin B specific activity for cleavage of Z-Arg-Arg-AMC was compared to that of cathepsins L, K, S, and V at pHs 7.2, 5.5, and 4.6. (c) Z-Phe-Arg-AMC: cathepsin B specific activity for cleavage of Z-Phe-Arg-AMC was compared to that of cathepsins L, K, S, and V at pHs 7.2, 5.5, and 4.6. All data are shown as the mean ± SD (*n* = 3).

Z-Nle-Lys-Arg-AMC specifically detected cathepsin B activity and not cathepsins L, K, S, V, and X activities evaluated at pHs 4.6, 5.5, and 7.2 (Figure 5 and Supporting Information Figure S4). In contrast, Z-Arg-Arg-AMC was not specific for cathepsin B since this substrate was cleaved by cathepsins L and V. The Z-Phe-Arg-AMC substrate was cleaved by cathepsin L with greater activity than that for cathepsin B; this substrate was also cleaved by cathepsins K and V. These data demonstrate Z-Nle-Lys-Arg-AMC as a specific substrate for cathepsin B.

### Specific Cathepsin B Activity in Neuronal and Glial Cells Assessed by the Z-Nle-Lys-Arg-AMC Substrate

**Figure 6:**
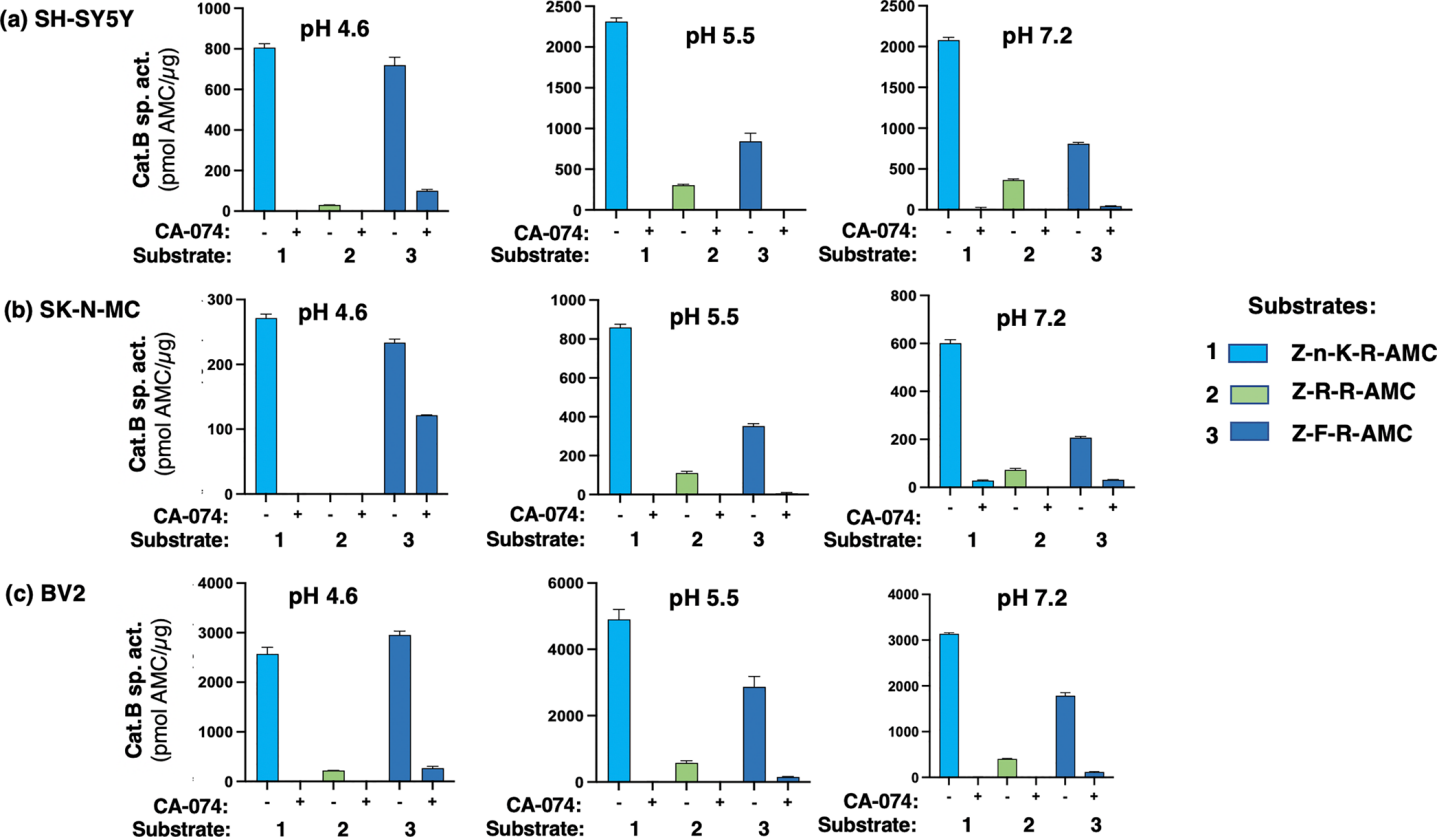
Cathepsin B assessed as CA-074-sensitive activity in neuroblastoma and microglial cells assessed with Z-Nle-Lys-Arg-AMC compared to Z-Arg-Arg-AMC and Z-Phe-Arg-AMC. Proteolytic activity of cathepsin B and other cysteine cathepsins were measured by fluorogenic assays of cell homogenates incubated with Z-Nle-Lys-Arg-AMC, Z-Arg-Arg-AMC, and Z-Phe-Arg-AMC substrates (60 μM for each substrate) at pHs 4.6, 5.5, and 7.2 with or without pre-incubation with CA-074, a potent and selective cathepsin B inhibitor. Cells assessed consisted of (a) human neuroblastoma SH-SY5Y, (b) human neuroblastoma SK-N-MC, and (c) mouse microglia BV2. Results display specific activity in the absence and presence of CA-074 which indicates cathepsin B specific activity. Data show cathepsin B activity as pmol AMC/μg enzyme protein (30 min incubation) as the mean ± SD (*n* = 3).

To evaluate the novel substrate Z-Nle-Lys-Arg-AMC in a complex biological sample that contains other proteases and proteins, the assay of cathepsin B activity in homogenates of human neuroblastoma cells (SHSY-5Y and SK-N-MC neuroblastoma) and mouse microglial cells (BV2 microglia) compared the use of the Z-Nle-Lys-Arg-AMC substrate to that of Z-Arg-Arg-AMC and Z-Phe-Arg-AMC substrates. Cathepsin B activity was assessed as CA-074 sensitive activity (Figure 6), ensuring that the observed activity was inhibited by the specific CA-074 inhibitor of cathepsin B.^58,59^ For the three cell types, the highest specific activity of cathepsin B was observed at pH 5.5 compared to pH 4.6 and pH 7.2 (Supporting Information Figure S5). Cathepsin B is present in situ within the pH 5.5 environment of secretory vesicles that produce peptide neurotransmitters.^60^ The two neuroblastoma cell lines showed similar specific activities of cathepsin B. The microglial cells displayed high cathepsin B specific activity that was 2 to 4 times higher than that of the neuroblastoma cells depending on the pH. Notably, the Z-Arg-Arg-AMC substrate detected none or little activity at pH 4.6 in the three cell types, whereas the Z-Nle-Lys-Arg-AMC substrate detected robust cathepsin B activity.

To assess the prediction that mouse cathepsin B resembles human cathepsin B for substrate preferences as demonstrated by the cellular assays, purified recombinant mouse cathepsin B was evaluated with the Z-Nle-Lys-Arg-AMC and Z-Arg-Arg-AMC substrates, as well as the Z-Phe-Arg-AMC substrate at pHs 4.6, 5.5, and 7.2 (Supporting Information Figure S6). The highest specific activity of mouse cathepsin B was observed with Z-Nle-Lys-Arg-AMC among the three substrates. Z-Arg-Arg-AMC-cleaving activity of cathepsin B was low. A moderate specific activity was observed with Z-Phe-Arg-AMC. These activities of mouse cathepsin B with the three substrates were represented by *k*_cat_/*K*_m_ kinetic values, showing high catalytic efficiency for the Z-Nle-Lys-Arg-AMC substrate, modest catalytic efficiency for Z-Phe-Arg-AMC, and low catalytic efficiency for the Z-Arg-Arg-AMC substrate (Supporting Information Figure S6). These findings demonstrate Z-Nle-Lys-Arg-AMC as an excellent substrate for mouse cathepsin B as well as for human cathepsin B.

### Higher Abundance of Cathepsin B Protein in Microglia Compared to Neuroblastoma Cells Represented by Cathepsin B Activities

To assess whether the higher cathepsin B specific activity in microglia compared to that in neuroblastoma cells may be related to the greater levels of cathepsin B protein, the relative levels of cathepsin B protein in these cells were evaluated by proteomics MS (Table 2). Label-free proteomics identified and quantitated the relative levels of cysteine cathepsins. The level of cathepsin B in microglial cells was about 3-fold greater than in the two neuroblastoma cell types, thus, correlating with the observation of an average of about 3 times greater cathepsin B activity in microglia compared to neuroblastoma cells.

**Table 2:** Cysteine Cathepsins in Neuroblastoma and Microglial Cells Assessed by Proteomics^a^

cell type	cysteine cathepsin	identification by nano-LC–MS/MS	quantifiable, peak area × 10^6^
neuroblastoma SH-SY5Y (human)	cathepsin B	+	8.97 ± 2.69
	cathepsin C	+	(not quantifiable)
neuroblastoma SK-N-MC (human)	cathepsin B	+	6.11 ± 3.84
	cathepsin C	+	(not quantifiable)
microglia BV2 (mouse)	cathepsin B	+	28.08 ± 10.47
	cathepsin C	+	1.07 ± 0.46
	cathepsin K	+	17.51 ± 5.48
	cathepsin L	+	7.62 ± 2.11
	cathepsin X	+	21.80 ± 3.66

a Cysteine cathepsin proteins present in neuroblastoma SH-SY5Y and SK-N-MC cells, and in microglia BV2 cells, were assessed by quantitative label-free proteomics. Proteomics identified the cathepsins, and relative quantitation is provided for those that met the criteria for quantification (described in Materials and Experimental Details).

It is interesting to observe that the microglial cells contained higher levels of multiple cysteine cathepsins compared to the neuroblastoma cells. The microglial cells contained robust levels of cathepsins B, D, and X, along with cathepsins A, K, L, and C. In the SH-SY5Y neuroblastoma cells, only two cathepsins, B and D, were detected; in the SKNMC neuroblastoma cells, cathepsins B and D were abundant and low levels of cathepsin C were detected.

These findings illustrate the cell type-specific differences in the expression levels of cathepsin B and related cysteine cathepsins. Notably, our newly developed substrate, Z-Nle-Lys-Arg-AMC, effectively detects the relative levels of cathepsin B activity. These results contribute to defining the variability of cathepsin B activity among different cell types and highlight the utility of Z-Nle-Lys-Arg-AMC as a tool for assessing cathepsin B specific activity in biological systems.

## Discussion

The prominent functions of cathepsin B in health and disease occur in acidic lysosomes and at the neutral pH of cytosol, nuclei, and extracellular locations. Notably, cathepsin B displays different substrate cleavage properties at the acidic pH compared to neutral pH conditions.^45−47^ It is, therefore, necessary to develop specific substrates for cathepsin B that specifically measure its proteolytic activity over broad pH ranges. Evaluation by this study of current substrates used to monitor cathepsin B activity, consisting of Z-Phe-Arg-AMC and Z-Arg-Arg-AMC, found that they lack specificity since they are cleaved by other cysteine cathepsins; furthermore, Z-Arg-Arg-AMC does not optimally assess cathepsin B activity at acidic pH. Therefore, the purpose of this study was to conduct in-depth substrate cleavage profiles of cathepsin B compared to other cysteine cathepsins K, L, S, V, and X for designing specific fluorogenic substrates that can monitor cathepsin B proteolytic activity at acidic to neutral pH conditions. Cleavage profiles of these proteases were assessed by MSP-MS that analyzed cleavages of peptide library components at pH 4.6 and pH 7.2. Analysis of the preferred and non-preferred residues at the P3, P2, and P1 positions adjacent to the P1–P1′ cleavage sites predicted the tripeptide Z-Nle-Lys-Arg-AMC as a preferred substrate for cathepsin B. Significantly, Z-Nle-Lys-Arg-AMC displayed the advantageous properties of measuring high cathepsin B specific activity over acidic to neutral pHs and was specific for cathepsin B over other cysteine cathepsins. Z-Nle-Lys-Arg-AMC specifically measured cathepsin B activity in neuronal and glial cells which were consistent with relative abundances of cathepsin B protein. These findings validate Z-Nle-Lys-Arg-AMC as a novel substrate that specifically monitors cathepsin B activity over a broad pH range.

Substrate cleavage profiling analysis of cathepsin B and cysteine cathepsins conducted by MSP-MS, at pH 4.6 and pH 7.2, revealed each protease’s distinct pattern of preferred amino acids at P4 to P4′ positions of the P1–P1′ cleavage sites of the peptide library. We focused on the P3 to P1 residue preferences for designing Z-peptide-AMC substrates that utilize non-prime site residues of cleavage sites. Comparisons showed that cathepsin B displayed unique preferences over other cysteine cathepsins, consisting of Nle at the P3 position, Lys at the P2 position, and Arg at the P3 position at both acidic and neutral pHs. This evaluation formed the basis for the design of Z-Nle-Lys-Arg-AMC as a specific cathepsin B substrate. Indeed, the assessment of Z-Nle-Lys-Arg-AMC with a series of Z-peptide-AMC substrates indicated that Nle was important over other substitutions consisting of Leu, Tyr, Phe, Ala, Trp, Gly, or Val. Dipeptides containing dibasic sequence combinations of Lys and Arg were poor compared to Z-Nle-Lys-Arg-AMC as the substrate. The pH profile of Z-Nle-Lys-Arg-AMC detected cathepsin B of high specific activity at pH 4.5 to pH 7.5, which covers the biological pH range of cathepsin B within acidic lysosomes to modestly acidic secretory vesicles and the neutral cytosol, nuclei, and extracellular locations.

The high catalytic activity and specificity of Z-Nle-Lys-Arg-AMC were based on the preference of cathepsin B for P3 and P2 residues adjacent to the P1–P1′ cleavage site. When compared to the Z-dipeptide-AMC substrates, the Z-tripeptide-AMC variants with inclusion of Nle as the P3 residue enhances cathepsin B specificity and catalytic efficiency, as shown by the higher specific activities for Z-Nle-Lys-Arg-AMC and Z-Nle-Arg-Arg-AMC compared to their dipeptide variants Z-Lys-Arg-AMC and Z-Arg-Arg-AMC, respectively. Notably, Val or Gly at P3 prevented any turnover of the substrate, suggesting that the P3 residue interacting with the enzyme S3 pocket plays a key role in substrate binding even when favorable amino acids are present at other positions. Additionally, at the P2 position, cathepsin B preferred Lys and Arg basic residues residues at both acidic pH 4.6 and neutral pH 7.2.

In neuronal and glial cells, cathepsin B activity was measured with high specific activity with the novel substrate Z-Nle-Lys-Arg-AMC. Cathepsin B activity was confirmed by inhibition by CA-074, a specific inhibitor of cathepsin B,^52,53^ indicating CA-074-sensitive activity. While Z-Nle-Lys-Arg-AMC was highly sensitive for monitoring cathepsin B activity, Z-Arg-Arg-AMC could monitor only low cathepsin B activity. The Z-Phe-Arg-AMC substrate detected cathepsin B as well as other cysteine cathepsin activity and, thus, was not specific for cathepsin B.

Z-Nle-Lys-Arg-AMC holds promise for evaluation of cathepsin B in various cellular and disease conditions. For example, cathepsin B is secreted by cancer cells into the tumor microenvironment^41−43^ where cathepsin B activity can be monitored by Z-Nle-Lys-Arg-AMC. Furthermore, bacterial infection promoted cellular cathepsin B activity at a neutral pH environment^61^ and, thus, the Z-Nle-Lys-Arg-AMC can be valuable for monitoring cathepsin B at neutral pH cellular environments in infectious diseases. The sensitivity of this substrate is particularly advantageous for detecting low concentrations of cathepsin B in diverse biological and pathological systems that would otherwise not be detected with the traditionally used substrates. Therefore, use of this substrate may enable a more comprehensive understanding of the role of cathepsin B in human diseases and biological systems.

Findings of this study show that the novel Z-Nle-Lys-Arg-AMC substrate is specific for cathepsin B over other cysteine cathepsins and is a sensitive substrate to monitor cathepsin B activity over a broad pH range from acidic to neutral conditions. This substrate is advantageous over other routinely used substrates for cathepsin B which are non-specific or unable to detect the enzyme’s activity at low to high pH conditions. Z-Nle-Lys-Arg-AMC detects cathepsin B in human and mouse species cell types and thus will be useful for clinical cathepsin B biomarker studies as well as for mouse models of human disease conditions.

## Supporting Information Available

Cathepsins B, K, L, S, and V cleavage profiling analyzed by MSP-MS and at various pHs, evaluation of cathepsin X to cleave Z-peptide-AMC substrates, specific cathepsin B activity monitored with novel Z-Nle-Lys-Arg-AMC substrates in human neuroblastoma and mouse microglial cells, mouse cathepsin B displaying pH-dependent activity with the substrates, and kinetic values of human cathepsin B assessed with various substrates (PDF)

## Notes

All mass spectrometry data have been deposited into massive.ucsd.edu under identifier MSV000090043 and proteomeXchange under identifier PXD035641.
