# Supplementary material for: Distinct Cleavage Properties of Cathepsin B Compared to Cysteine Cathepsins Enable the Design and Validation of a Specific Substrate for Cathepsin B over a Broad pH Range
Source: Biochemistry. 2023 Jul 17;62(15):2289–300. doi: 10.1021/acs.biochem.3c00139 (PMC10399199; doi:10.1021/acs.biochem.3c00139)
Supplement: Supplementary file 1 — bi3c00139_si_001.pdf [file bi3c00139_si_001.pdf]

Supplemental Information

Distinct cleavage properties of cathepsin B (CTSB) compared to cysteine cathepsins provide design and validation of a specific substrate for CTSB over a broad pH range

Michael C. Yoon[1, 2], Von Phan[1, 2], Sonia Podvin[1], Charles Mosier[1],
Anthony J. O'Donoghue[1], and Vivian Hook[1,2,3*]

[1]Skaggs School of Pharmacy and Pharmaceutical Sciences, University of California, San Diego, La Jolla, CA 92093 USA;
[2]Biomedical Sciences Graduate Program, University of California, San Diego, La Jolla, CA 92093;
[3]Department of Neurosciences and Department of Pharmacology, School of Medicine, University of California, San Diego, La Jolla, CA 92093 USA

* Corresponding Author:
Dr. Vivian Hook, Skaggs School of Pharmacy and Pharmaceutical Sciences, University of California San Diego, 9500 Gilman Dr. MC0657, La Jolla, CA 92093-0657, email vhook@ucsd.edu

Supplemental Materials consist of:

Figures S1 to S6

Table S1

Figure S1. Cathepsins B, K, L, S, and V cleavage profiling analyzed by MSP-MS. Volcano plots of cathepsin B (panel a), cathepsin K (panel b), cathepsin L (panel c), cathepsin S (panel d), cathepsin V (panel e), and cathepsin X (panel f) peptide cleavages from MSP-MS data show the $\log_2$ ratios of relative quantities of peptide products generated by each of these cathepsins compared to no enzyme activity controls, illustrated by $-\log_{10}$ p values. Peptide products generated with at least an 8-fold change above controls and with $p < 0.05$ were analyzed for the frequencies of amino acid residues at the P4−P4′ positions of the P1−↓P1′ cleavage site in the heatmaps and iceLogos depicted in Figures 2 and 3.

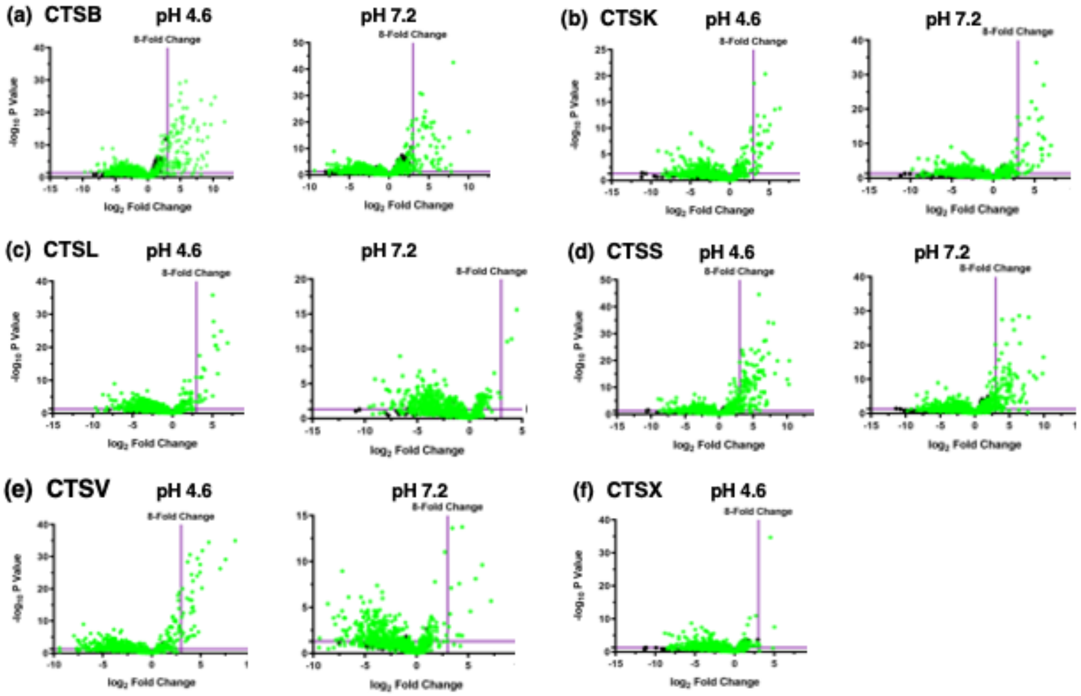

Figure S2. Cathepsin B and cathepsin K cleavage profiles at pH 4.6 and pH 7.2 assessed for preferred residues at P4 to P4' positions adjacent to P1-P1' cleavage sites.

(a) Cathepsin B cleavage profiles at pH 4.6 and pH 7.2. The preference of cathepsin B cleavages for amino acid residues at each of the P4 to P4' positions adjacent to the P1-P1' cleavage site was deduced from MSP-MS data cleavage profiling data. Z-scores provided quantitation of preferred (green shades) and non-preferred (yellow shades) residues illustrated in a heat map. The z-scores were utilized for iceLogo illustration of the primary preferred residues at the P4 to P4' positions shown above the mid-line, with non-preferred residues shown below the mid-line. The purple letters indicate significance of $p < 0.05$, and the black letters indicate $p < 0.3$.

(b) Cathepsin K cleavage profiles at pH 4.6 and pH 7.2. The preference of cathepsin K cleavages for residues at P4 to P4' positions was defined from MSP-MS data, indicated by z-scores shown in a heat map for preferred residues (green shades) and non-preferred residues (yellow shades). The primary preferred residues, based on z-scores, were plotted in iceLogo illustrations. The purple letters indicate significance of $p < 0.05$, and the black letters indicate $p < 0.3$.

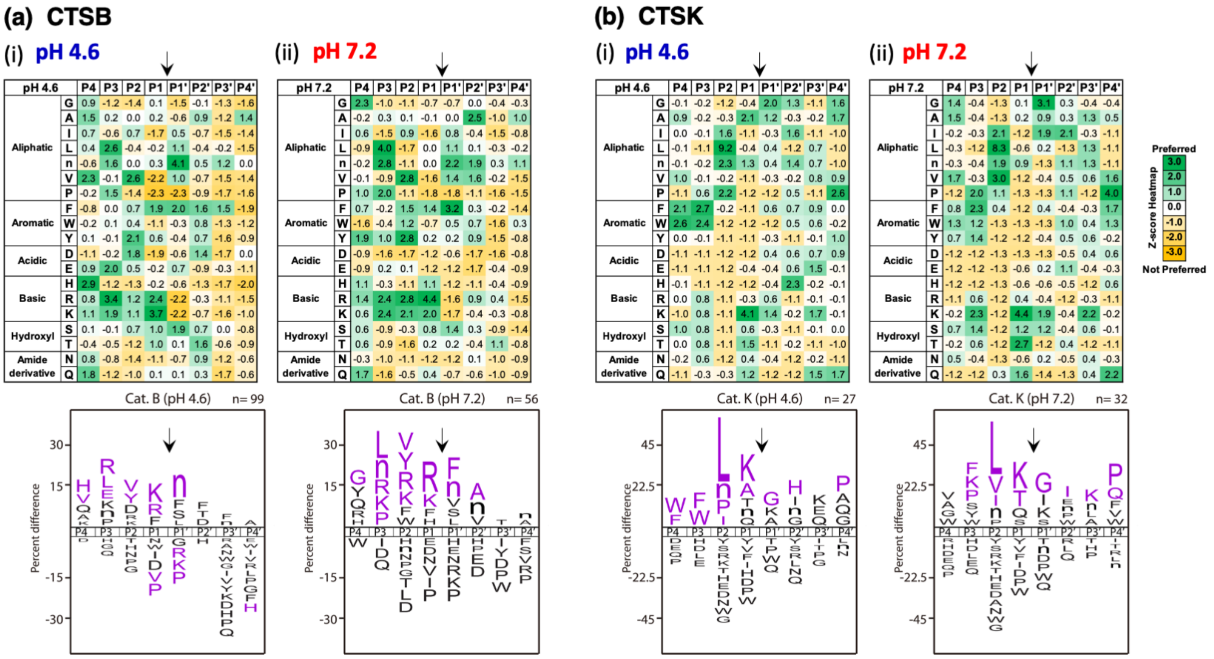

Figure S3. Cathepsins L, S, and V cleavage profiles at pH 4.6 and pH 7.2 assessed for preferred residues at P4 to P4' positions.

The preferences of each cathepsin protease for residues at P4 to P4' positions were defined by MSP-MS data, and indicated z-scores for preferred residues (green shades) and non-preferred residues (yellow shades). IceLogo illustrated the main preferred residues (above the line) and preferred residues with significance of $p < 0.05$ are shown in purple letters.

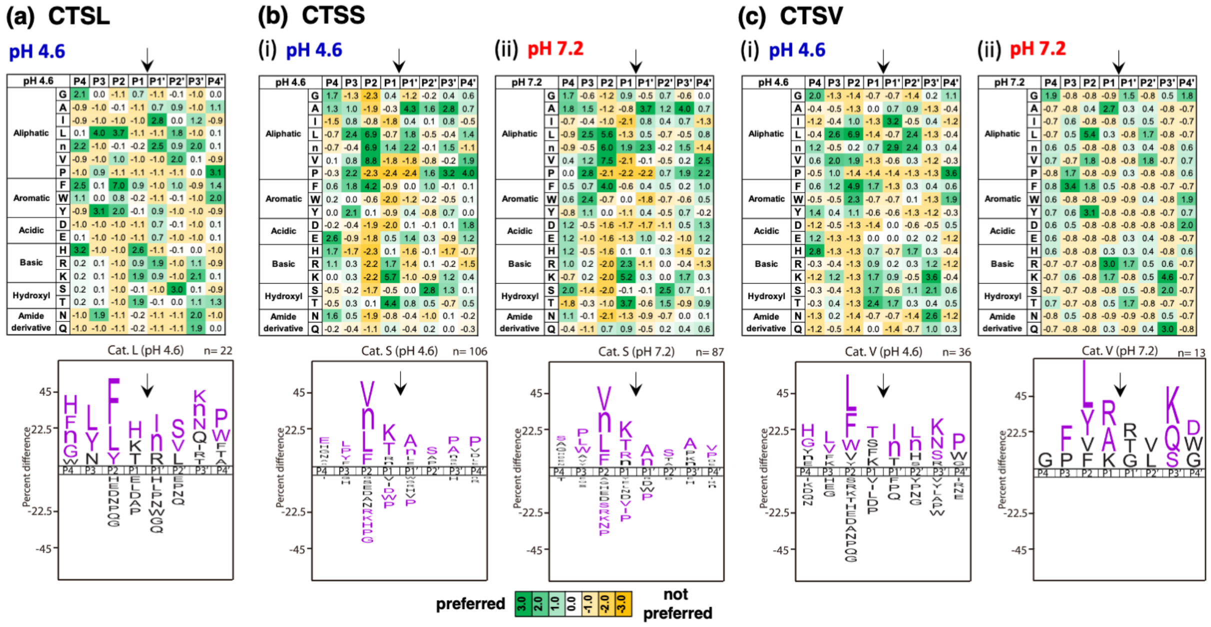

Figure S4. Evaluation of cathepsin X to cleave Z-peptide-AMC substrates of Z-Nle-Lys-Arg-AMC, Z-Phe-Arg-AMC, and Z-Arg-Arg-AMC.

Cathepsin X activity is illustrated with its standard substrate Mca-RPPGFSAFK(Dnp)-OH, showing activity at pH 4.6 and pH 5.5 (but not at pH 7.2). Cathepsin X was assessed for proteolytic cleavage of the Z-Nle-Lys-Arg-AMC, Z-Phe-Arg-AMC, or Z-Arg-Arg-AMC substrates (40 μM substrate concentrations) at the three pHs tested of pH 4.6, 5.5, and 7.2. Cathepsin X did not display cleavage of these fluorogenic substrates.

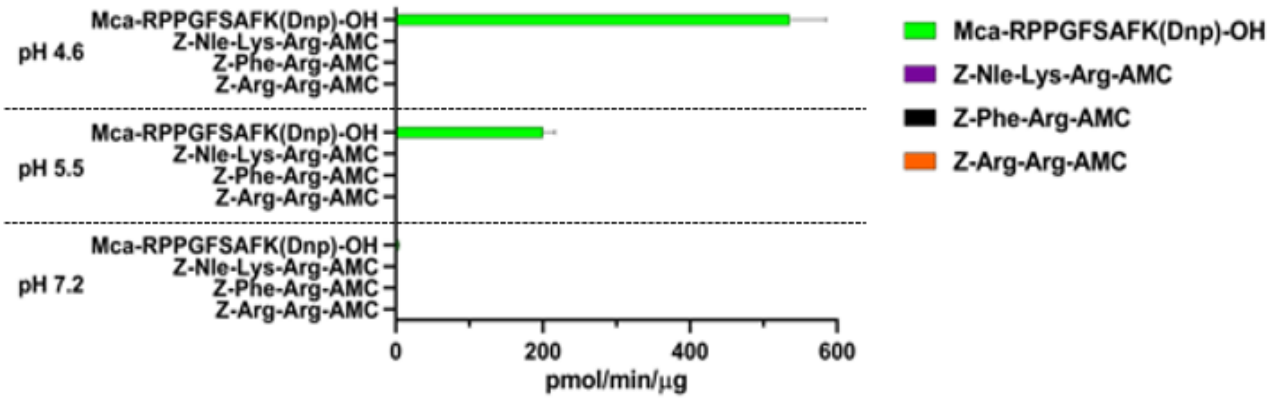

Figure S5. Specific cathepsin B activity monitored with novel Z-Nle-Lys-Arg-AMC substrate in human neuroblastoma and mouse microglia cells. Cathepsin B activity in cell homogenates from human neuroblastoma SHSY-5Y cells (panel a), human neuroblastoma SK-N-MC cells (panel b), and mouse microglia BV2 cells (panel c) was assessed with the substrates Z-Nle-Lys-Arg-AMC, Z-Arg-Arg-AMC, and Z-Phe-Arg-AMC (60 μM) at pH 4.6, 5.5, and 7.2. Data show cathepsin B activity as pmol AMC/μg enzyme protein (30 min incubation) as the mean ± SD (n=3). Significance is indicated by *p < 0.05 (student's t-test) comparing proteolytic activity with the of substrates Z-Arg-Arg-AMC and Z-Phe-Arg-AMC compared to Z-Nle-Lys-Arg-AMC.

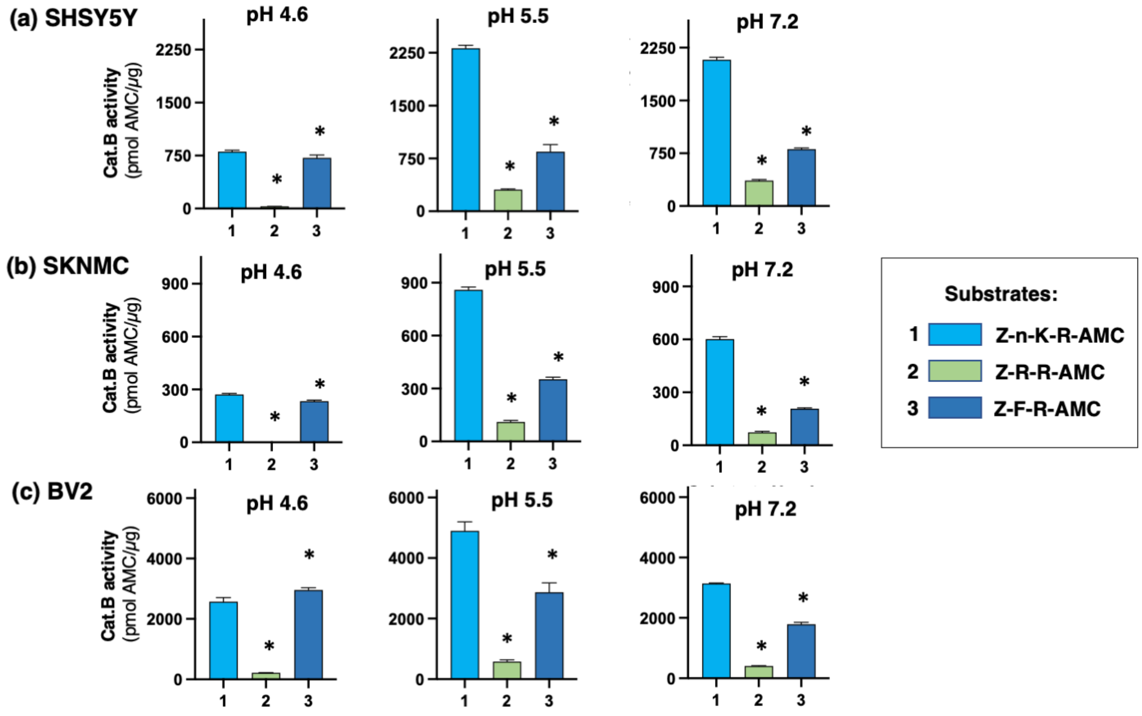

Figure S6. Mouse cathepsin B displays pH-dependent activity with the substrates Z-Nle-Arg-Lys-AMC, Z-Arg-Arg-AMC, and Z-Phe-Arg-AMC.

(a) pH-dependence of mouse cathepsin B activity with Z-Nle-Lys-Arg-AMC compared to Z-Arg-Arg-AMC and Z-Phe-Arg-AMC substrates. Cathepsin B specific activity with the three different substrates (40 μM) were assessed at pH 4.6, 5.5, and 7.2. Data points are shown as the mean ± SD (n = 3).

(b) Kinetic k_{cat}/K_m values for cathepsin B activity with the substrates Z-Nle-Lys-Arg, Z-Arg-Arg-AMC and Z-Phe-Arg-AMC. k_{cat}/K_m values for each of the substrate(s at pH 4.6 and pH 7.2 were calculated for mouse cathepsin B as described in the methods. (The asterisk indicates that the k_{cat} /K_m was determined from the linear slope of the curve since the curve did not plateau out.)

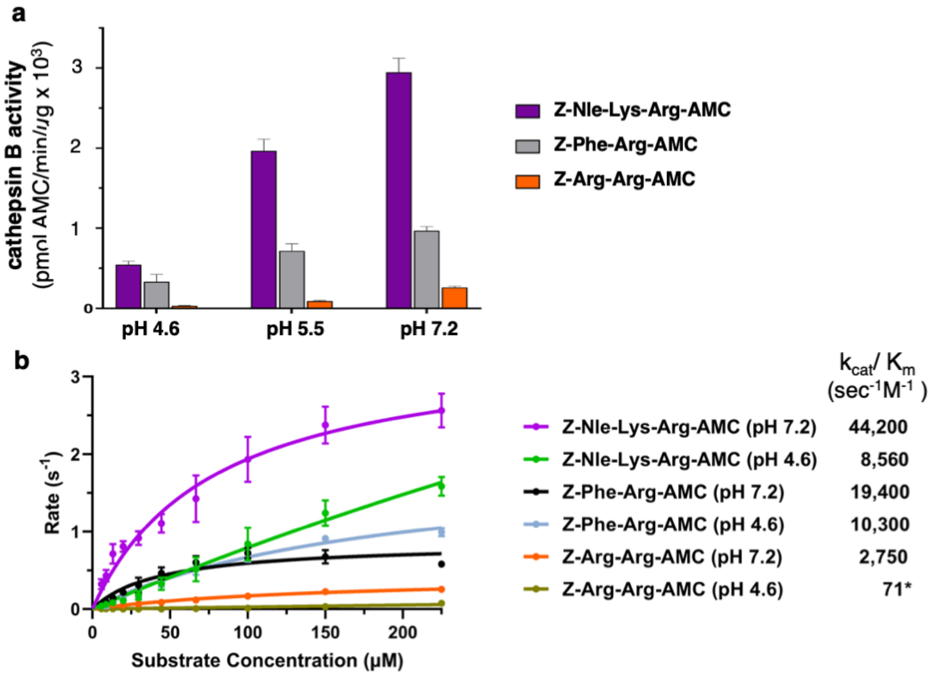

Table S1. Kinetic values of human cathepsin B assessed with substrates Z-Nle-Lys-Arg-AMC, Z-Arg-Arg-AMC, and Z-Phe-Arg AMC at pH 4.6 and 7.2.

Z-Nle-Lys-Arg-AMC

	z-nKR-AMC (pH 4.6)	z-nKR-AMC (pH 7.2)
k_{cat} (s^{-1})	18.5 ± 8.5	7.4 ± 1.0
K_m (μM)	1254 ± 649	216 ± 50
k_{cat}/K_m ($s^{-1}M^{-1}$)	14761	34035

Z-Arg-Arg-AMC

	z-RR-AMC (pH 4.6)	z-RR-AMC (pH 7.2)
k_{cat} (s^{-1})	n.d.*	2.8 ± 0.2
K_m (μM)	n.d.*	395 ± 42
k_{cat}/K_m ($s^{-1}M^{-1}$)	930*	7065

Z-Phe-Arg-AMC

	z-FR-AMC (pH 4.6)	z-FR-AMC (pH 7.2)
k_{cat} (s^{-1})	1.5 ± 0.1	1.0 ± 0.1
K_m (μM)	55.2 ± 7.4	29.9 ± 5.0
k_{cat}/K_m ($s^{-1}M^{-1}$)	26885	33645

Kinetic values were determined from three independent determinations assessed for Michaelis-Menten kinetics with standard deviation (conducted using GraphPad Prism).

n.d. = not determined.
